# Determinants of Microdamage in Elderly Human Vertebral Trabecular Bone

**DOI:** 10.1371/journal.pone.0055232

**Published:** 2013-02-15

**Authors:** Hélène Follet, Delphine Farlay, Yohann Bala, Stéphanie Viguet-Carrin, Evelyne Gineyts, Brigitte Burt-Pichat, Julien Wegrzyn, Pierre Delmas, Georges Boivin, Roland Chapurlat

**Affiliations:** 1 INSERM, UMR 1033, Lyon, France; 2 Université de Lyon, UMR 1033, Lyon, France; 3 Département de Chirurgie Orthopédique, Pavillon T, Hôpital Edouard Herriot, Lyon, France; University of Notre Dame, United States of America

## Abstract

Previous studies have shown that microdamage accumulates in bone as a result of physiological loading and occurs naturally in human trabecular bone. The purpose of this study was to determine the factors associated with pre-existing microdamage in human vertebral trabecular bone, namely age, architecture, hardness, mineral and organic matrix. Trabecular bone cores were collected from human L2 vertebrae (n = 53) from donors 54–95 years of age (22 men and 30 women, 1 unknown) and previous cited parameters were evaluated. Collagen cross-link content (PYD, DPD, PEN and % of collagen) was measured on surrounding trabecular bone. We found that determinants of microdamage were mostly the age of donors, architecture, mineral characteristics and mature enzymatic cross-links. Moreover, linear microcracks were mostly associated with the bone matrix characteristics whereas diffuse damage was associated with architecture. We conclude that linear and diffuse types of microdamage seemed to have different determinants, with age being critical for both types.

## Introduction

Fatigue microdamage accumulates in bone as a result of physiological loading. [1], [2], [3], [4] Several studies have reported microdamage in human trabecular bone occurring naturally. [5], [6], [7], [8], [9], [10] Increased microdamage is associated with decreased bone strength in vitro, and thus may play a role in fragility fractures. [11] Whereas microdamage appears to increase with age [6], [12] and decreased with trabecular bone volume [13], few studies have examined whether bone microarchitecture and/or characteristics of the bone matrix, such as the degree of mineralization, mineral maturity, and collagen crosslink profile are associated with the accumulation of microdamage in human trabecular bone [14], [15].

In cortical bone, microcracks are observed within the interstitial bone or in interstitial bone intersecting with osteonal cement lines, and are arrested by osteons. [12], [16], [17] In cortical bone, microdamage appears to initiate within highly mineralized regions in cortical bone, [18] which is consistent with the linear elastic fracture mechanics theory [19], [20].

Nucleation of mineral occurs in the ‘hole’ regions of the collagen arrays and apatite crystals develop in length along the collagen long axes and in width along channels within the collagen sheets. In such a two-phase structure, a microcrack would most likely be a break or fissure not only in the mineral matrix, but also in organic matrix. [21], [22]. Thus, regarding mineral matrix, the degree of mineralization, cement lines or crystal size [23] may have a specific role in the initiation of microdamage, as they are mainly located in old interstitial regions which are more mineralized. [16], [17] Indeed, changes in the morphology of the mineral crystal itself may affect the bone mechanical properties and microdamage accumulation. [24], [25] For example, in human femur, crystallinity explained up to 48% of the variation in monotonic mechanical properties, and up to 64% in fatigue properties. [26] In addition to the mineral phase, the type and amount of collagen cross-links by impacting strength and stability between collagen fibers may be also associated with changes in microdamage. [27], [28] Recent studies showed that the extent of advanced glycation endproducts (AGEs) were negatively associated not only with mechanical parameters [29], [30], [31] and microarchitecture [32] but also with microdamage. [33], [34] In contrast, mature enzymatic cross-links seemed to be associated with less microdamage [33].

Altogether, the relationships between *in vivo* microdamage and the intrinsic properties of human trabecular bone remain ill-defined. In particular, little is known about what factors are specifically associated with linear and/or diffuse microdamage. Thus, the primary aims of the present study were to determine whether the degree and heterogeneity of mineralization, the mineral and organic characteristics, the microhardness and the collagen cross-links are related to the amount and type of pre-existing microdamage (ie: no damage created for this study) independently of age and trabecular bone volume in human vertebral trabecular bone from older donors.

## Materials and Methods

### Ethics Statement

Human bone samples were obtained from French body donation to science program (Laboratory of Anatomy, Faculty of Medicine Lyon Est, University of Lyon, France).

### Specimen Preparation

L2 vertebrae were taken from 53, recently deceased Caucasians donors (22 men, 30 women), mean age of 78.3±9.9 years, with a range 54–95 years of age. The sex of one subject and the age of two subjects were not known. Bone samples were wrapped in gauze soaked with saline to keep them wet, then stored at −20°C. Vertebrae were screened using medial-lateral and anterior-posterior high-definition X-rays (Faxitron X-Ray, Lincolnshire IL, USA) to exclude prevalent fracture and significant bone diseases (e.g., metastasis, Paget's disease, osteochondritis). No additional information regarding donor disease status or medication history was available. Each vertebra was sectioned in half using an Isomet Buehler 4000 microsaw (Buehler GmbH, Düsseldorf, Germany). One hemi vertebra was dedicated to mechanical tests and the bone surrounding the cores was collected for collagen cross-links quantification by HPLC [10]; the second hemi vertebra was dedicated to the detection of pre-existing microdamage and mineral analysis. The hemi vertebrae for microdamage evaluation were bulk stained for 11 days at room temperature in 0.005 M xylenol orange (Sigma-Aldrich Corp., St. Louis, MO, USA) based in 70% ethanol. In each hemi vertebrae, a cylindrical trabecular specimen (8.25 mm diameter, 25 mm length) was removed in the supero-inferior direction from the anterior quadrant using a diamond tipped coring tool. The end plate of each vertebra was removed, as previously described. [10], [35] Trabecular bone volume (BV/TV, %), trabecular thickness (Tb.Th, µm), trabecular number (Tb.N, 1/mm), trabecular separation (Tb.Sp, µm), connectivity density (Conn.D, 1/mm^3^), degree of anisotropy (DA), and structure model index (SMI), which reflects the rod- versus plate-like nature of the structure, of the excised cores were assessed by microcomputed tomography, using an isotropic voxel size of 20 microns, energy (70 kVp), current (177 µA), integration time (200 ms) and a threshold corresponding to a mineral density around 0.35 g.cm^3^ (µCT40, Scanco Medical AG, Brüttisellen, Switzerland). Structural indices of trabecular bone analysis were calculated using distance transformation (filling structure with spheres) without assuming anything about the shape of the bone (i.e. without plate model assumption). BV/TV was based on counting voxels.

### Microdamage analysis

**Figure 1:**
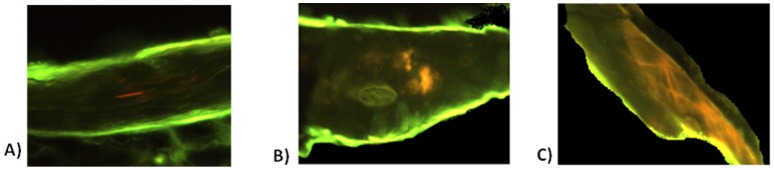
Pictures obtained under fluorescent light of microdamage in orange: A) Linear microcrack, B) Diffuse damage, C) Few cross-hatching microdamage were found.

Trabecular cores for detection of microdamage were embedded in methylmethacrylate and cut parallel to the long axis to obtain at least three non-contiguous, parallel, 100±5 µm-thick sections for microdamage evaluation, as previously described. [35], [36] Specimen were bulk stained with xylenol orange and general non-specific staining, morphologically different from microcracks, were easily identifiable. Three sections per specimen were measured using fluorescence (excitation/emission wavelength of 440–570/610 nm) microscopy at ×200 magnification and morphometry software (Bone Morpho; Explora Nova, La Rochelle, France). Microdamage appeared in orange under fluorescence light. [35], [36] Microdamage was categorized and quantified as linear microcracks or diffuse damage (only few cross-hatching, which were counted as diffuse damage, Figure 1). Outcome assessments included the linear crack density, defined as the number of linear microcracks (Cr.N, #) per bone area (Cr.Dn, #/mm^2^), and the diffuse damage density, defined as the number of diffuse damage regions per bone area (Dx.Dn, #/mm^2^). Length was expressed as mean linear crack length (Cr.Le, µm)^1^, whereas length and area density were expressed as linear length density (Cr.Le.Dn, µm/mm^2^)^2^ and diffuse damage area density defined as diffuse damage area per bone area (Dx.Ar.Dn, %).

NB: 1- Mean linear crack length is the mean of total length of linear cracks measured on three sections divided by the total number of linear cracks.

2- Crack length density is the mean crack length divided by the bone volume (BV) measured, by histomorphometry, on the same three sections: Cr.Le.Dn =  Cr.Le/BV.

### Mineralization Measurements

The mean degree of mineralization and heterogeneity of mineralization were assessed using quantitative microradiography on 100±1 µm thick sections, as previously described. [37], [38], [39] Outcomes included the mean degree of mineralization of bone (DMB, g/cm^3^) and the heterogeneity index expressed as the mean of the widths at half-maximum height (HI, g/cm^3^) measured on the individual DMB curves.

### Hardness Analysis

Microhardness (Hv, kg/mm^2^) was measured on polished remaining embedded blocks using a Vickers indenter with a 25 µg load for 10 seconds, as previously described [38].

### Mineral at Crystal Level, and Organic Matrix Analysis by FTIRM

**Figure 2:**
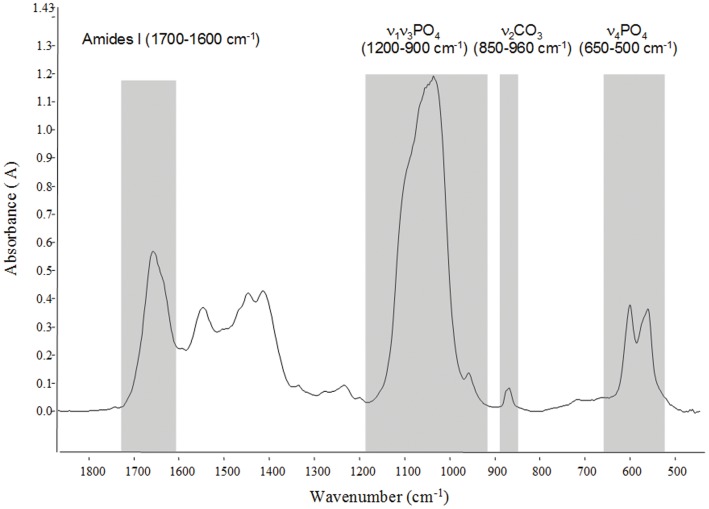
Infrared spectrum obtained from vertebral trabecular bone (man, 79 year-old). Spectrum is expressed as wave number (cm^−1^) which represent the vibration modes (amide I, phosphate, carbonate).

Each infrared spectra spectrum (Figure 2), performed on 2-µm-thick sections, was collected from an area of 30×100 µm^2^ at a spectral resolution of 4 cm^−1^ and 50 scans were performed by spectrum. [40] Twenty spectra were analyzed per sample. The following variables were determined:

The mineral crystallinity index (CI) corresponds to both crystal size and perfection [40], the mineralization index (MI) is the area ratio of the bands of mineral matrix over organic matrix [41], mineral maturity (MM) was calculated as the area ratio of the apatitic phosphate over non apatitic phosphate reflecting also the age of mineral [40], carbonation was calculated as integrated area ratios ν_2_CO_3_ (862–894 cm^−1^) over ν_1_ν_3_PO_4_ (910–1184 cm^−1^), representing the quantity of carbonates incorporated into the bone mineral, and collagen maturity (CM) was calculated as the ratio of organic matrix bands (1660/1690 cm^−1^ area ratio) [42] reflecting the evolution of the secondary structure of collagen with the mineralization [43].

### Collagen Cross-links Analysis

The central part of the vertebral body around the core, containing exclusively trabecular bone, was retained for biochemical analysis. Cross-links measurements were carried out as previously described. [32], [44] We assessed the content of both enzymatic nonreducible mature cross-links PYD and DPD, and the PEN induced by non-enzymatic induced, normalized to the total amount of collagen.

### Statistical Analysis

Data are reported as mean values, standard deviations, ranges, median and interquartile range. As some variables were not normally distributed even after transformation, we used non-parametric tests. No significant differences between men and women were observed, consequently men and women were pooled for correlation analyses. The relationships between parameters were assessed by Spearman correlation coefficient (r). We used partial spearman correlation to test whether associations between parameters were significant after accounting for the contribution of BV/TV or age. All tests were two-tailed, and significance defined as p≤0.05. All statistical analyses were performed using statistical analyses software (R & SPSS 16).

## Results

### Association between Age, Microdamage and its Determinants

#### Descriptive statistics are found in Table 1

**Figure 3:**
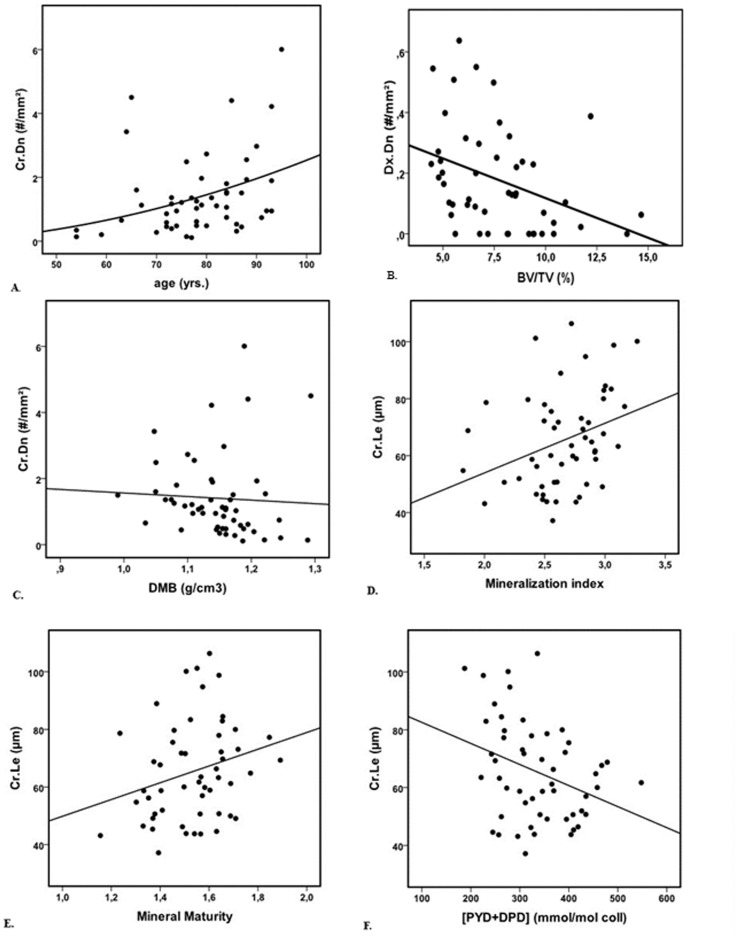
Bivariate Scattergrams. A) Age of donor vs Crack density – r = 0.36, p = 0.010, B) BV/TV vs Diffuse damage density, r = −0.42, p = 0.003, C) Degree of mineralization vs crack density, r = −0.31, p = 0.02, D) Mineralization index vs Crack length, r = 0.36, p = 0.008, E) Mineral maturity vs Crack length, r = 0.30, p = 0.03, F) Mature cross-links vs Crack length, r = −0.30, p = 0.03.

Microdamage (crack density – Figure 3a, crack length density, and diffuse damage area density), Tb.Sp, SMI, the ratio PYD/DPD and crystallinity index increased with age, whereas carbonation, BV/TV, Tb.N and DA decreased with age (Table 2). Except the ratio PYD/DPD, collagen cross-link characteristics, mineralization parameters and microhardness were not related to age (Table 3). After adjustment for BV/TV, crack length density, PYD/DPD, carbonation, mineral maturity and crystallinity index remained significantly correlated with age (Table 4).

**Table 1:** Descriptive statistics, mean ± SD, range, median and interquartile range (IQR).

	n	Mean±SD	Range		Median	IQR
Age (yrs)	51	78.3±9.9	54	95	78	12.8
*–FTIRM parameters*	53					
Mineralization Index		2.66±0.32	1.82	3.27	2.64	0.411
Mineral Maturity		1.54±0.15	1.16	1.89	1.56	0.24
Carbonation		0.007±0.001	0.006	0.009	0.007	0.001
Collagen Maturity		4.03±0.46	2.91	5.30	3.98	0.57
Crystallinity Index		25.23±0.81	23.39	27.28	25.31	1.14
*–Architecture*	51					
BV/TV (%)		7.68±2.42	4.43	14.66	7.33	3.59
Tb.Th (µm)		137±18	102	187	133	28.3
Tb.Sp (µm)		1161±189	878	1932	1125	222
Tb.N (1/mm)		0.84±0.12	0.51	1.11	0.84	0.16
Connectivity density		2.03±0.80	0.74	4.32	1.87	1.14
SMI		1.77±0.49	0.33	2.52	1.80	0.74
DA		1.68±0.20	1.28	2.36	1.64	0.21
*–Mineralization*	53					
DMB (g/cm^3^)		1.147±0.061	0.99	1.293	1.155	0.076
HI (g/cm^3^)		0.287±0.087	0.122	0.525	0.274	0.111
*–Hardness*	53					
Hv (Kg/cm^2^)		45.97±1.65	42.37	48.91	45.74	2.83
*–Microdamage*	53					
Cr.N (#)		22±24	1	109	15	20.3
Dx.N (#)		2.54±2.7	0	11	2.0	2.5
Cr.Dn (#/mm^2^)		1.408±1.24	0.114	6.012	1.109	1.132
Dx.Dn (#/mm^2^)		0.172±0.168	0	0.637	0.128	0.216
Cr.Le (µm)		65.37±17.15	37.1	106.37	63.24	26.79
Cr.Le.Dn (µm/mm^2^)		93.1±92.8	7.68	456.3	69.35	68.0
Dx.Ar.Dn (%)		0.034±0.065	0	0.344	0.013	0.024
*–Collagen X-Links*	53					
PYD (mmol/mol coll)		232.1±61	126	406	225	91
DPD (mmol/mol coll)		103.6±25.9	61	192	103	32
PYD/DPD		2.30±0.65	1.398	5.65	2.184	0.635
PYD+DPD(mmol/mol coll)		335.7±78	187	548	128	91
PEN (mmol/mol coll)		20.85±9.41	6.19	54.5	18.4	11.71
Collagen (%)		22.77±2.49	15.30	28.74	23.27	3.197

**Table 2:** Bivariate spearman coefficient correlation (r_sp_) between microdamage, age, architecture, mineralization degree, microhardness, mineral at crystal level, organic matrix and collagen cross-links.

	Age	Cr.Dn	Dx.Dn	Cr.Le	Cr.Le.Dn	Dx.Ar.Dn
	r_sp_	r_sp_	r_sp_	r_sp_	r_sp_	r_sp_
Age		**0.357** *	0.232	0.02	**0.353** *	**0.345** *
***–FTIRM parameters***						
Mineralization	0.223	−0.106	−0.043	**0.358** **	0.021	−0.007
Mineral Maturity	0.144	0.018	−0.11	**0.298** *	0.109	−0.091
Carbonation	−**0.353** *	0.053	0.082	−0.079	−0.002	−0.009
Collagen Maturity	0.045	0.025	0.034	−0.039	0.006	−0.045
Crystallinity Index	**0.336** *	0.074	−0.093	0.22	0.152	−0.049
***–Architecture***						
BV/TV	−**0.574** **	−0.20	−**0.417** **	0.160	−0.151	−**0.379** **
Tb.Th	0.10	0.025	0.032	**0.456** **	0.157	0.034
Tb.Sp	**0.36** *	−0.20	**0.424** **	−0.052	0.005	**0.321** *
Tb.N	−**0.34** *	0.005	−**0.431** **	−0.070	−0.018	−**0.317** *
Conn density	−0.199	−0.187	−**0.356** *	−0.102	−0.195	−0.194
SMI	**0.513** **	0.085	0.213	−0.057	0.086	0.237
DA	−**0.348** *	−0.043	0.216	**0.277** *	0.008	0.064
***–Mineralization***						
DMB	0.030	−**0.314** *	−0.172	**0.319** *	−0.237	−0.108
HI	−0.033	−0.058	−0.075	−0.071	−0.097	−0.033
–***Hardness***						
Hv	0.136	−0.035	−0.087	0.233	0.024	−0.100
***–Collagen X-Links***						
PYD	0.272	**0.326** *	0.164	−**0.30** *	0.229	0.101
DPD	0.119	0.057	0.197	−**0.275** *	−0.032	0.213
PYD/DPD	**0.307** *	**0.326** *	−0.011	−0.12	**0.279** *	−0.005
PYD+DPD	0.222	0.269	0.21	−**0.296** *	0.170	0.134
PEN	0.071	0.001	−0.063	0.074	0.036	−0.065
Collagen	−0.131	−0.261	−0.040	0.122	−0.180	0.008

* p<0.05,

** p<0.01.

**Table 3:** Spearman partial coefficient after adjustment for age; dependent variables shown along the top and independent variables along the side of the table.

	Cr.Dn	Dx.Dn	Cr.Le	Cr.Le.Dn	Dx.Ar.Dn
	r	r	r	r	r
***–FTIRM*** ***parameters***					
MineralizationIndex	−0.197	−0.092	**0.352** **	−0.071	−0.087
Mineral Maturity	−0.043	−0.179	**0.292** *	0.054	−0.165
Carbonation	0.195	0.141	−0.04	0.153	0.120
Collagen Maturity	−0.005	−0.024	−0.067	−0.009	−0.087
Crystallinity Index	−0.054	−0.191	0.212	0.034	−0.195
***–Architecture***					
BV/TV	−0.011	−**0.389** **	0.246	0.064	−0.254
Tb.Th	−0.005	0.04	**0.451** **	0.114	0.031
Tb.Sp	−0.165	**0.393** **	0.025	−0.159	0.232
Tb.N	0.139	−**0.398** **	−0.043	0.131	−0.230
Conn density	−0.123	−**0.316** *	−0.09	−0.119	−0.127
SMI	−0.093	0.173	−0.07	−0.108	0.109
DA	0.074	**0.297** *	**0.328** *	0.147	0.199
***–Mineralization***					
DMB	−**0.355** **	−0.194	**0.327** *	−0.270	−0.124
HI	−0.056	−0.077	−0.090	−0.095	−0.019
***–Hardness***					
Hv	−0.096	−0.128	0.243	−0.032	−0.165
***–Collagen X-Links***					
PYD	0.242	0.061	−**0.315** *	0.151	−0.017
DPD	0.0012	0.130	−**0.281** *	−0.082	0.160
PYD/DPD	0.24	−0.12	−0.10	0.21	−0.14
PYD+DPD	0.197	0.120	−**0.309** *	0.104	0.038
PEN	−0.04	−0.094	0.09	0.005	−0.111
Collagen	−0.224	0.034	0.090	−0.166	0.079

* p<0.05,

** p<0.01.

**Table 4:** Spearman partial coefficient after adjustment for BV/TV; dependent variables shown along the top and independent variables along the side of the table.

	Age	Cr.Dn	Dx.Dn	Cr.Le	Cr.Le.Dn	Dx.Ar.Dn
	r	r	r	r	r	r
Age		0.268	−0.056	0.194	**0.311** *	0.132
***–FTIRM parameters***						
Mineralization	0.240	−0.151	−0.089	**0.418** **	−0.008	−0.028
Mineral Maturity	**0.333** *	0.021	−0.084	**0.334** *	0.110	−0.062
Carbonation	−**0.326** *	−0.018	0.195	−0.149	0.060	0.080
Collagen Maturity	0.106	−0.0176	0.047	0.001	−0.043	−0.072
Crystallinity Index	**0.350** *	0.0286	−0.160	0.256	0.117	−0.122
***–Architecture***						
Tb.Th	0.240	0.045	0.129	**0.452** ***	0.167	0.111
Tb.Sp	0.046	−0.169	0.242	0.216	−0.103	0.165
Tb.N	−0.037	0.140	−0.262	−0.222	0.079	−0.168
Conn density	0.084	−0.146	−0.213	−0.214	−0.168	−0.060
SMI	0.217	−0.067	−0.165	0.115	−0.010	−0.024
DA	−0.161	0.042	**0.431** *	0.232	0.078	0.249
***–Mineralization***						
DMB	0.205	−0.269 BL	−0.049	0.258	−0.204	−0.011
HI	−0.121	−0.083	−0.157	−0.035	−0.118	−0.094
***–Hardness***						
Hv	0.093	−0.046	−0.154	0.246	0.028	−0.133
***–Collagen X-Links***						
PYD	0.1648	0.266 BL	0.045	−0.235	0.180	−0.026
DPD	0.003	−0.011	0.116	−0.223	−0.095	0.135
PYD/DPD	**0.301** *	**0.327** *	−0.064	−0.103	**0.285** *	−0.60
PYD+DPD	0.109	0.207	0.109	−0.233	0.116	0.020
PEN	0.129	0.026	−0.002	0.030	0.050	−0.060
Collagen	−0.011	−0.229	0.025	0.110	−0.137	0.122

* p<0.05,

** p<0.01.

*** p<0.001.

### Association between Bone Volume Fraction, Microarchitecture and Microdamage

Linear crack density was not associated with BV/TV. Linear crack length was positively associated with trabecular thickness (Tb.Th) and the degree of anisotropy (DA) (Table 2), even after adjusting for age (Table 3). After adjusting for BV/TV, Cr.Le remained strongly associated with Tb.Th (Table 4). In comparison, diffuse damage density was negatively correlated with BV/TV (Figure 3b), connectivity density and Tb.N, and positively correlated with Tb.Sp. After adjusting for age, diffuse damage density remained associated with BV/TV, Tb.Sp, Tb.N and connectivity, and association appeared with DA. After adjusting for BV/TV, all these associations disappeared, except for DA, which was positively correlated with damage density (Table 4). Diffuse damage area density was negatively correlated with BV/TV and Tb.N, and positively with Tb.Sp. (Table 2). After adjusting for BV/TV or age, all these associations disappeared (Table 3, Table 4).

### Association between Mineral Traits and Microdamage

Crack length was positively correlated with mineralization index, mineral maturity and DMB, whereas crack density was negatively correlated with DMB (Table 2, Figure 3c). After adjusting for age, these correlations remained significant (Table 3). After adjusting for BV/TV, only mineralization index and mineral maturity remained significantly associated with crack length (Table 4, Figure 3d,e) even if it was borderline for DMB (p = 0.056). Carbonation and crystallinity index were not correlated to microdamage, neither before nor after adjusting for age or BV/TV. Neither heterogeneity index (HI) nor the microhardness (Hv) was associated with microdamage in vertebral trabecular bone.

### Association between Collagen Cross-links, Organic Matrix Properties, and Microdamage

Crack density was positively correlated with PYD, (PYD/DPD), and tended to decrease with the percentage of collagen. Crack length was also negatively associated with PYD, DPD and (PYD+DPD), (Table 2, Figure 3f). After adjusting for age, only the relations between PYD, DPD, (PYD+DPD) and crack length remained significant (Table 3), whereas after adjusting for BV/TV, only the PYD/DPD ratio remained significantly associated to crack density and crack length density (Table 4). Crack length density was only correlated with the PYD/DPD ratio. Collagen maturity was not correlated with microdamage, even after adjusting for age or BV/TV. The non-enzymatic glycation product, PEN, was not associated with any parameters of microdamage.

## Discussion

In this study, we found that microdamage in human vertebral trabecular bone was associated with age, microarchitecture, mineral characteristics and mature enzymatic cross-links, with different factors associated with linear versus diffuse damage.

### Determinants of Linear Microcracks

Our finding that microdamage increases with age is in agreement with prior data reporting that linear microcracks (Cr.Dn, Cr.Le and Cr.Le.Dn) in trabecular bone from the femoral head, [11], [13] the neck, [6] and vertebrae [9], [35] increase exponentially with the age of the donors. [6], [7], [9], [11], [12], [13], [35], [45] In contrast with our previous study in only 23 vertebrae, [35] but similarly to work of Fazzalari et al [6], in the current study, microarchitecture was not associated with linear crack density. Compared to Arlot et al [35], statistical difference is found in the crack density (same age, BV/TV, crack length), the measurement of damage area (in the current study, when no damage was found, a value of “zero” was indicated contrary to previous study where a “none” value was indicated) and the number of samples (23 compared to 53 presently). But the main difference is found in the architectural measurement of trabecular bone. In the previous study [35], structural parameters (BV/TV, Tb.Th, Tb.Sp, Tb.N) were based on triangularization^1^ of surface (ie: one more interpolation step in comparison to VOX, based on counting voxels). In present study, we used the called “Direct” measurement (DT)^2^, based on distance transformation, which is more accurate. In prior study, Cr.Dn was linked with respectively TRI-BV/TV (r = −0.51, p = 0.017), TRI-Tb.N (r = −0.54, p = 0.012) and TRI-Tb.Sp (r = 0.53, p = 0.013). Using the direct measurement (DT), as in the current study, (for the same subset of samples), those previous associations does not remain, except for BV/TV: Cr.Dn and, respectively, Vox-BV/TV (r = −0.51, p = 0.018), indeed, DT-Tb.N (r = −0.31, p = 0.17) and DT-Tb.Sp (r = 0.32, p = 0.15). Furthermore, if the direct measurement was used for the previous study, crack length was also associated to the direct trabecular thickness.

NB: 1- Triangularization or TRI corresponds to traditional 2D histomorphometry, but this plate-model assumption leads to a bias in most cases. Scanco® recommends to use DT-Tb.N, DT-Tb,Sp for truly 3D results.

2- Direct measurement or DT indices are calculated using distance transformation (filling structure with spheres) without assuming anything about the shape of the bone (i.e. without plate model assumption).

The way of analysis the data is also essential. Indeed, depending of the analytical model used, Yeni et al., [46] who also studied human vertebral trabecular bone, found two different trends. A power law relationship between Cr. Dn and BV/TV was significant (r^2^ = 0.14, p = 0.025). However, a three-parameter exponential fit was not significant for the relationship between Cr. Dn and BV/TV (p>0.07). [46] There are too few papers on damaged aged human vertebrae to accurately compare with literature.

Average length of linear microcracks was associated with mineralization index, degree of mineralization and mineral maturity. The more mature the mineral, the longer the cracks. However, the crystallinity index was not associated with crack length. Linear microcrack density was not associated with any of the variables measured by FTIRM, suggesting that mineral maturity and/or mineralization does not influence microdamage accumulation in trabecular bone. [47] However, the degree of mineralization was inversely associated with Cr.Dn, and positively associated with Cr.Le, meaning the more mineralized the bone was, the fewer but longer the cracks were, consistently with the positive association between elastic modulus and crack length. [10] The ensuing discrepancy between the mineralization index and DMB might be explained. Indeed, while quantitative microradiography provides results reflecting the entire bone surface of the sample, FTIRM reflects a smallest proportion of analyzed area. Therefore, the number of measurements in trabecular bone did not cover the entire bone surface, explaining the discrepancy obtained between DMB and mineralization index. It has been suggested that the more bone was mineralized, the lower the plastic deformation was and more cracks were initiated. [48] The level of mineralization might be an important factor influencing the cracks morphology. [48] Degree of mineralization and mineral maturity do not influence number or length the cracks in the same way. This finding leads to the conclusion that mineral quantity rather than mineral characteristics (maturity or crystallinity) should be used to explain cracks number.

Recent *in vitro* [49] and *ex vivo* studies [33], [34] show that AGEs altered the extent of accumulated microdamage in bone tissue. Nevertheless, no association was found between PEN content and microdamage parameters, but an unexpected relationship between mature enzymatic cross-links and crack density was noted. Indeed, we found the same association between mature enzymatic cross-links and cracks density already than Saito et al [34] in cortical rib from dogs treated for 3 years with bisphosphonate. These discrepancies may be explained by the fact that microdamage may be site specific. Also, the collagen characteristic is bulk assessment (<10 mg of bone) compared to microdamage, a very localized process (thin sections). However an interesting association between mature enzymatic cross-link content and crack length was found suggesting that these cross-links, by providing strength and stability between collagen fibers, may avoid crack propagation in bone tissue. This result confirms that enzymatic cross-linking in bone is a favorable process. Lastly, collagen maturity, measured by FTIRM, was not related to microdamage whereas the collagen content, measured by HPLC, showed a negative trend with linear cracks density. This suggested that lower collagen content may be associated with numerous microcracks. Nevertheless, the orientation of the collagen fiber [50], the sacrificial bonds [51] may also participate to the propagation of microdamage and alter bone quality.

### Determinants of Diffuse Damage

We found that diffuse damage in human vertebral trabecular bone was associated with age, and microarchitecture. The surface density of diffuse damage was positively associated with age, conversely to Vashishth et al. [8] who found that age did not influence the accumulation of diffuse damage in men or women. [8] However, this is partly explained by the mean age of their female population (56 years-olds) near the onset of menopause, and associated with high turnover rates. In our study, diffuse damage was only associated with microarchitecture, but not a with either characteristics of the mineral matrix nor the variables measured by FTIRM [47].

### Relationship between Microcracks and Local Mechanical Properties Linked to Mineral Phase

Wang et al. showed that nanoindentation hardness was a very good predictor of bone tissue elastic modulus for both normal and osteoporotic bone tissues. [52] In another study, the same authors discussed the fact that postfailure properties of hard tissue had strong effects on bone microdamage morphology and the rate of change in apparent mechanical properties. [53] In our study, no relationship was found between hardness and microdamage, or between DMB and collagen maturity. Those hardness measurements were done at the tissue level (global level), and not at the local level of the microdamage itself. Thus, more precise measurements of hardness should be done around the microcracks to better understand the association between contact hardness and microdamage [54].

Our study has some limitations. An important limitation to the discussion of our results is that we have no information regarding medical history of persons, and therefore we cannot rule out the presence of diseases or medications that may have influenced our outcome variables. However, the anatomical specimens were radiographed to exclude bone diseases or tumors, and bone histology allowed to exclude specimens with osteomalacia. We acknowledge that many of our study subjects were old so our results are only representative of an elderly population. This group, however, is most susceptible to fragility fractures. We are conscious that some donors may have received an osteoporosis therapy (i.e. estrogen or bisphosphonate) which may alter the relationships with microdamage. For example, bisphosphonate has an effect on the crystal characteristics as shown in BSUs of similar DMB and mineral maturity, the crystallinity was decreased in patients long-term treated with alendronate. [55] This was also shown for zoledronate, when crystallinity was measured between tetracycline labels (same chronology in both groups), it was lower in patients treated for 3 years once yearly. Moreover, another bisphosphonate (risedronate) has been associated with changes in collagen maturity assessed by FTIRM. [56] Furthermore, we did not quantify the reducible cross-links which may contribute to skeletal fragility and we only analyzed pentosidine, which explained 40% of the variance in bulk fluorescent AGE and comprise only a portion of total fluorescent AGEs that accumulate with tissue age in bone [33].

Our study also has a number of strengths. First, we have analyzed a relatively large number of sample, and only one sample per donor (compared to other studies using cadaveric tissues) comprising old donors whose age distribution reflected the population in which the majority of fragility fractures occur. Furthermore, this is among the few studies to assess mineral characteristics, collagen cross-links, and microdamage analyses on the same vertebral body.

In conclusion, trabecular microdamage increases with age, but this association is driven, at least in part, by declining BV/TV. We also found that linear and diffuse damage have different determinants. Linear microcracks are mostly influenced by the bone matrix quality whereas diffuse damage seems to be influenced by bone microarchitecture. The second finding is that, within the linear microcracks, there are also different factors that influence the density and the length of cracks.
